# Customized Minimally Invasive Protocols for the Clinical and Microbiological Management of the Oral Microbiota

**DOI:** 10.3390/microorganisms10040675

**Published:** 2022-03-22

**Authors:** Andrea Scribante, Andrea Butera, Mario Alovisi

**Affiliations:** 1 Unit of Orthodontics and Pediatric Dentistry, Section of Dentistry, Department of Clinical, Surgical, Diagnostic and Pediatric Sciences, University of Pavia, 27100 Pavia, Italy; 2 Unit of Dental Hygiene, Section of Dentistry, Department of Clinical, Surgical, Diagnostic and Pediatric Sciences, University of Pavia, 27100 Pavia, Italy; 3 Department of Surgical Sciences, Dental School, University of Turin, 0121 Turin, Italy

In recent years, the personalization of periodontal clinical practice has led to the study of protocols with a proactive approach. These protocols limit the chemical pharmacological action over time as much as possible and maintain a balanced oral microbiota, while educating the patient in correcting home management and ensuring optimal compliance. This approach can reduce inflammatory indices, leading to the possibility of organizing minimally invasive operative sessions working on healthy tissues [1]. The modified one-stage full-mouth disinfection allows the reviewing of the standard protocols by means of quadrant treatment, providing the patient with all the knowledge to reduce the battery charge at home, through a correct removal of bacterial plaque with toothbrushes and interdental subsidies and inflammation combined with natural substances to accelerate the healing processes. Minimally invasive instrumentation has the purpose of reducing the incidence of soft tissue reorganization, acting only on the risk factors, and rebalancing the bacterial biota present within the gingival sulcus and crevicular fluid. Moreover, it also maintains the unchanged percentage of adherent gingiva and the gingival margin in healthy patients with systemic diseases like diabetes [2]. To emphasize the minimally invasive approach, guaranteeing a proactive action to balance oral dysbiosis, in the last decade the focus has been placed on the use of probiotics [3] and paraprobiotics [4]. If these are used for a quarterly period, by taking chewing gum, toothpastes, and mousses, they have the ability to reduce the bacterial load. In vivo studies with the microbiological analysis of the bacterial load have seen a statistically significant reduction of the orange complex. The orange complex includes bacteria which, as characteristics, have the ability to form a bond between the early colonizers and the highly pathogenic bacteria of the red complex. Orange complex bacteria are responsible for the progressive loss of attachment and the increase in pocket depth. Through their metabolism, these bacteria also create the living conditions for the strictly anaerobic bacteria of the red complex and their colonization of the periodontal sulcus and counts of *Prevotella intermedia, Campylobacter rectus,* and *Fusobacterium nucleatum*, which are the bacteria responsible for the progression and chronicization of periodontal disease. They have the capacity to decrease the incidence of bacteria present (both orange and red complexes, formed by the four oral bacteria, *Aggregatibacter actinomycetemcomitans*, *Tannerella forsythensis, Porphyromonas gingivalis, and Treponema denticola,* responsible for the severe clinical manifestation of periodontal disease), to reduce the indication of periodontal disease, and to act as a double action in lowering the carioreceptive pathogenic load [5]. The effectiveness of both glycine [6] and erythritol-based powders [7] should not be excluded to maintain a minimally invasive standard, respecting the hard and the soft oral tissues and reducing the bacterial load of the red complex, with a higher percentage of reductions in *Porphyromonas gingivalis* in periodontal and implantological patients. The ozone therapy falls within the range of dental multidisciplinarity, oxygenating the hard and soft tissues. It exerts its antiseptic and antibacterial action, reducing the incidence of mucositis and peri-implantitis, periodontal disease, lesions to the mucous membranes of the oral cavity, and both surgical and endodontic post-operative edema [8,9]. Ozone, by its division into three oxygen molecules, has the ability to penetrate inside the DNA and RNA of bacteria and viruses and destroy their structure and is a guaranteed way of reducing healing times by accelerating the healing action. All these protocols have the ability to integrate the bacterial load, increasing the percentage of commensal bacteria, avoiding the resetting of the oral microbiota and the use of chemical pharmacological substances, in order to improve the clinical practice of the operator and that of the patients at home [10].

The new techniques recently published are postbiotics, thanks to their antioxidant action, of food origin, they accelerate the healing processes by reducing the battery charge both on periodontal and implant tissues. All these proactive, professional, and home-based approaches, aimed at improving the clinical activity of professionals and the quality of patients’ home practices, are welcome in our Special Issue “Customized Minimally Invasive Protocols for the Clinical and Microbiological Management of the Oral Microbiota”, in order to have an increasingly minimally invasive approach and to safeguard the well-being of patients.

## Data Availability

Not applicable.
